# Exosomal YB-1 facilitates ovarian restoration by MALAT1/miR-211-5p/FOXO_3_ axis

**DOI:** 10.1007/s10565-024-09871-8

**Published:** 2024-05-03

**Authors:** Mengxue Zhang, Jie Xing, Shijie Zhao, Minjun Lu, Yueqin Liu, Li Lin, Wujiang Gao, Lu Chen, Wenxin Li, Junyu Shang, Jiamin Zhou, Xinming Yin, Xiaolan Zhu

**Affiliations:** 1 Reproductive Center, The Fourth Affiliated Hospital of Jiangsu University, 20 Zhengdong Road, Zhenjiang, Jiangsu 212001 People’s Republic of China; 2 Center for Reproductive Medicine, Affiliated Hospital of Nantong University, Nantong, Jiangsu People’s Republic of China; 3 Institute of Reproductive Sciences, Jiangsu University, Zhenjiang, 212001 Jiangsu People’s Republic of China; 4 Department of Central Laboratory, The Fourth Affiliated Hospital of Jiangsu University, Zhenjiang, People’s Republic of China; 5 Department of Obstetrics and Gynecology, Chengdu Women’s and Children’s Central Hospital, School of Medicine, University of Electronic Science and Technology of China, Chengdu, People’s Republic of China; 6 Department of Obstetrics and Gynecology, The Fourth Hospital of Changsha, Changsha, People’s Republic of China

**Keywords:** POF, sEVs, YB-1, MALAT1

## Abstract

Premature ovarian failure (POF) affects many adult women less than 40 years of age and leads to infertility. Mesenchymal stem cells-derived small extracellular vesicles (MSCs-sEVs) are attractive candidates for ovarian function restoration and folliculogenesis for POF due to their safety and efficacy, however, the key mediator in MSCs-sEVs that modulates this response and underlying mechanisms remains elusive. Herein, we reported that YB-1 protein was markedly downregulated in *vitro* and in *vivo* models of POF induced with H_2_O_2_ and CTX respectively, accompanied by granulosa cells (GCs) senescence phenotype. Notably, BMSCs-sEVs transplantation upregulated YB-1, attenuated oxidative damage-induced cellular senescence in GCs, and significantly improved the ovarian function of POF rats, but that was reversed by YB-1 depletion. Moreover, YB-1 showed an obvious decline in serum and GCs in POF patients. Mechanistically, YB-1 as an RNA-binding protein (RBP) physically interacted with a long non-coding RNA, MALAT1, and increased its stability, further, MALAT1 acted as a competing endogenous RNA (ceRNA) to elevate FOXO_3_ levels by sequestering miR-211-5p to prevent its degradation, leading to repair of ovarian function. In summary, we demonstrated that BMSCs-sEVs improve ovarian function by releasing YB-1, which mediates MALAT1/miR-211-5p/FOXO_3_ axis regulation, providing a possible therapeutic target for patients with POF.

**Supplementary Information:**

The online version contains supplementary material available at 10.1007/s10565-024-09871-8.

## Introduction

Premature Ovarian Failure (POF) is one of the common illnesses found in women causing 1% female infertility. It is biochemically characterized by amenorrhea with hypoestrogenic and hypergonadotropic conditions (Nippita and Baber 2007). Increasing evidence indicates oxidative damage-induced senescence and even apoptosis of granulosa cells (GCs), the critical part of a follicle, form the microenvironment for oocyte growth, and maturation, is a trigger of age-related follicular atresia and even ovarian dysfunction (Akogullari et al. 2018; Santoro 2003). Although hormone replacement therapy (HRT) can alleviate the symptoms of estrogen deficiency, no credible therapies other than oocyte donation have been found to increase fertility in women with POF (Torrealday et al. 2017).

Nevertheless, egg donation resources are scarce, and patients accepting donated eggs can never own their biological offspring. Thus, clinicians are searching for new treatments for POF, and Mesenchymal stem cells (MSCs) transplantation is a promising treatment, unfortunately, the limited amplification ability in *vitro*, possible immune response to allograft transplantation (Tauchmanovà et al. 2007), occlusion in microvasculature (Furlani et al. 2009), and potential tumorigenicity (Jeong et al. 2011) make it difficult to effectively reach target organizations and apply in clinical practice (Ankrum and Karp 2010; Phinney and Prockop 2007). Therefore, the development of cell-free therapies that circumvent cellular immunogenicity using secretome, cytokine, and sEVs derived from MSCs has been a growing research focus (L et al. 2019).

Unlike cells, the membrane vesicles, sEVs do not elicit acute immune rejection and are much smaller, have been considered a safer alternative approach to MSCs therapy (Toh et al. 2018). Our and others' researches indicated that MSCs-sEVs can recover ovarian function and folliculogenesis in chemotherapy-induced POF rats via inhibiting oxidative damage-induced cell apoptosis of GCs (Ding et al. 2020; Gao et al. 2023; Yang et al. 2020a; b), however, the therapeutic mechanisms of MSCs-sEVs action on POF are poorly understood and worth further exploring. Those cells that take up sEVs, also known as recipient cells, present some phenotypic changes due to the modulatory effects of functional molecules delivered from sEVs. Growing interest in the involvement of active proteins carried by sEVs in the modulation of aging (Chen et al. 2018; Luo et al. 2021). As one of the members that can be transmitted through exosomes, the RNA-binding protein (RBP) YB-1 is considered to be an oncogenic factor in many solid tumors by promoting cell proliferation (Guo et al. 2022; Jiang et al. 2022; Yin et al. 2022). Notably, YB-1 has been shown to be a cellular stress response factor that is essential for inhibiting premature cell failure in cultured cells in *vitro* (Lu et al. 2005) and considered to be a promising molecular candidatet for modulating cellular senescence (Xiao et al. 2020). Interestingly, we detected downregulation of YB-1 protein in ovarian tissue of chemotherapy-induced POF rats as well as in H_2_O_2_-induced POF cell model, which was reversed after MSCs-sEVs transplantation, therefore, we strongly questioned whether MSCs-sEVs could deliver YB-1 to reverse ovarian function and the potential mechanism.

By binding to RNA, YB-1 was involved in several posttranscriptional modulation of gene expression, including RNA splicing (Rapp et al. 2002), stability (Dhawan et al. 2012), localization (Kawaguchi et al. 2012; Wilhelm et al. 2000) and translation (Evdokimova et al. 2009). A variety of noncoding RNAs was found to be associated with YB-1. These RNAs correspond to thousands of distinct short YB-1-associated noncoding RNAs and their cognate processed YB-1-associated small RNAs that primarily match to the 5′or 3′ terminal regions, and were capable of affecting many fundamental biochemical pathways through interaction with the global regulator, YB-1. Therefore, to explore the potential target RNAs of YB-1, we used the RIP-seq data (GSE130781) and CLIP-seq data (GSE150925) of YB-1 as a guide to identify MALAT1, a long non-coding RNA (LncRNA) which was overexpressed in many tumors. Next, we demonstrated that YB-1 physically binds and stabilizes MALAT1, and miR-211-5p/FOXO_3_ was the direct target of MALAT1 by bioinformatics analysis and loss/gain-of function approaches. Thus, our study reports a new function of MSCs-sEVs secreted YB-1 in preventing oxidative stress-induced ovarian GCs injury during aging and the mechanisms of action.

## Materials and methods

### Cell culture and transfection

The human granulosa-like tumor cell line (KGN) used in this study was obtained from Ji he Biotechnology Co., LTD (Shanghai, China). The KGN was cultured in medium (C11995500BT, Gibco, USA) with 10% fetal bovine serum (FBS) (10270–106, Gibco, USA) and 1% penicillin–streptomycin (SV30010, Hyclone, USA) and grown in 5% CO_2_ atmosphere at 37 °C. The Plasmid YB-1 RNAi were obtained from Genechem (Shanghai, China). Si-MALAT1, miR-211-5p inhibitor, si-FOXO_3_ and their relative NC were obtained from GenePharma (Shanghai, China). Cells were plated into 6-well plates (TCP001006, Jet Bio-Fil, China) the day before transfection and then transfected with plasmids or siRNA using Lipofectamine 2000 reagent (11668019, Thermo Fisher Scientific, USA) at 70% or 50% cell fusion After 48 h, cells were used for further experiments.

### The establishment of the POF model in vitro

KGN were plated into 6-well plates 12 h prior to treatment. When approximately 30% cell confluence was reached, 100 μm of H_2_O_2_ was added directly for 24 h for induction of cellular senescence.

### Patient group and GCs acquisition

The serum samples and GCs utilized in this research were acquired from healthy volunteers and POF patients at the Fourth Affiliated Hospital of Jiangsu University. All patients and healthy volunteers were informed, and written consent was signed. Each group had 10 patient samples. The research protocol was approved by Jiangsu University Ethics Committee.

### Western blot analysis

After total proteins were harvested, a western blot was performed according to standard methods to detect protein expression. Briefly, total proteins were separated by SDS-PAGE (P1200, Solarbio, China) and then transferred to polyvinylidene difluoride membranes (1620184, Bio-Rad, USA). After blocking, the membranes were first imprinted with YB-1 (ab12148, Abcam, UK), P53(WL01919, Wanleibio, China), P21(WL0362, Wanleibio, China), FOXO_3_ (WL02891, Wanleibio, China) or GAPDH (ab9485, Abcam, UK) primary antibodies for 12 h at 4 °C followed by membrane wash and secondary antibodies (#14708, Cell Signaling Technology, USA) incubation for 1.5 h. The protein band signals were detected by ECL chemiluminescent substrate (E412-01, Vazyme, China) in ChemiDoc™ XRS + with Image Lab™ software (Bio-Rad, USA).

### Isolation and culture of BMSCs

The harvesting of BMSCs consisted of the following steps. First, bone marrow was harvested by flushing the tibia and femur of 5-week-old SD rats. Then, suspensions containing BMSCs were placed in α‐MEM (C12571500BT, Gibco, USA) containing 20% FBS and 1% penicillin–streptomycin cultured in a cell culture incubator. In the next step, the suspension containing unaffixed BMSCs was discarded after 73 h. Subsequently, the cells were passaged when they were 80–90% fused. Finally, BMSCs that were passaged for 3–7 generations were used for experiments or extraction of sEVs.

### SEVs isolation, quantification, and characterization

SEVs isolation was performed using an exosome isolation kit (UR52121, Umibio, China). After removing cell fragments, the supernatant was added exosome concentration solution (ESC) in the proportion of 4:1. After blending, the mixed liquid was placed at 4 °C for 2 h. Then, sEVs precipitation is separated out by centrifugation at 10000 × g for 1 h. Then, the PBS-washed sEVs were purified with an exosome purification filter at 3000 × g for 10 min at 4 °C. Lastly, the sEVs were stored at − 80 °C for further investigation. BCA protein assay kit (CW0014, CWBIO, China) was used to determine the protein concentration of sEVs. The marker protein CD9 (ab236630, Abcam, UK) was detected by western blot analysis. Transmission electron microscopy (TEM; H-7800, Hitachi, Japan) and nanosight tracking analysis (220-Twin, Particle Metrix, GER) were used to measure the appearance and size of sEVs.

### Measurement of intracellular ROS levels

The Kit (S0033M, Beyotime Biotechnology, China) for measuring intracellular ROS levels was purchased from Beyotime Biotechnolog. Cells and ovary tissues of different groups were treated with DCFH-DA diluted according to the instructions at 37 °C for at least 30 min and then rinsed gently with PBS for three times. Finally, the images were captured immediately under Leica DM4 B & DM6 B Upright Microscope (Leica, GER) and the image capture software was LAS X.

### Assay of cellular senescence (S-A-β-gal staining)

The Cell Senescence S-A-β-galactosidase Staining Kit (C0602, Beyotime, China) was employed to assess S-A-β-gal activity. The S-A-β-gal staining efficiency was determined as the proportion of positively stained cells to the sum of cells in a single field of view. The images were captured immediately under Leica DM4 B & DM6 B Upright Microscope and the image capture software was LAS Core.

### Real-time quantitative PCR (qPCR)

Treatment of cells with TRIZOL reagent (15596–026, Thermo Fisher Scientific, USA) is the method of total RNA isolation. cDNA was obtained with a reverse transcription kit (KR118, TIANGEN, China and B532451, Sangon, China) in S1000 Thermal Cycler (1852196, Bio-Rad, USA). The qPCR primers were customized at Sangon Biotech (Shanghai, China). qPCR was used to analyze targeted RNA expression by utilizing SYBR PCR kit (B639271 and B532461, Sangon, China) and was performed in 7500 fast real-time qPCR system (4351106, Thermo Fisher Scientific, USA). The relative expression of RNA was performed using the 2-△△Ct method. The primer sequences were displayed in Supplementary Information: Table S1.

### Data acquisition and analysis of LncRNA-MiRNA-mRNA network

YB-1-associated RIP-seq (GSE130781) and CLIP-seq (GSE150925) were collected from Gene Expression Omnibus (http://www.ncbi.nlm.nih.gov/geo/), and ceRNA network was predicted in the online website of Starbase (https://rnasysu.com/encori/).

### RNA immunoprecipitation (RIP) assay

The reagents used for the RIP experiments were EZ-Magna RIP kit (Millipore, Billerica, USA). The experimental steps were succinctly described as follows. First, RIP lysis buffer was applied to lysed HEK-293 cells. Second, PBS-washed magnetic beads were washed by RIP wash buffer, RNAs magnetic beads were conjugated with an anti-YB-1 antibody or negative control anti-IgG. Next, 100 μl cell lysate, 900 μl RIP buffer, and magnetic beads mixed continuously at 4 °C until overnight. Next, the magnetic beads combining YB-1-RNA were digested by proteinase K for 0.5 h at 55 °C. Then, the magnetic beads combining YB-1-RNA were treated with phenol and chloroform after a RIP buffer wash. Next, salt solution I, II, precipitate enhancer and anhydrous ethanol were added, and the RNA-containing liquid was placed at − 80 °C overnight. Finally, the RNA was obtained by centrifugation of the above liquid and dissolved in Dnase/Rnase-free ddH_2_O for qPCR analysis.

### Pull-down assay with biotinylated miRNA

HEK-293 cells were transfected with biotinylated miR-211-5p probe and the negative control probe customized at Genepharm (Shanghai, China). Then, cells were lysed with 550 μl lysis buffer containing protease inhibitor (78,430, Thermo Fisher Scientific, USA) and Rnase inhibitor (R0102, Beyotime, China). Cell lysates and Streptavidin-Dyna beads (65801D, Thermo Fisher Scientific, USA) were mixed overnight at 4 °C with constant rotation. Finally, RNA was extracted by adding 750 μl Trizol LS per sample and 250 μl water and then subjected to qPCR as explained above.

### Luciferase report assay

The Phusion Mutagenesis kit (F541, Thermo Fisher, USA) was used to mutate the binding sites. cDNAs that contained the wild-type sequences WT-FOXO_3_ or mutated binding sequencesor MUT-FOXO_3_ with miR-211-5p in FOXO_3_ 3′ UTR were cloned into the pGL4 luciferase reporter vector (Promega, USA). HEK-293 cells were co-transfected with the recombinant plasmid together with miR-211-5p mimics or mimics NC (synthesized from Genepharma, China). Then, the co-transfected cells were obtained in the Reporter Lysis Buffer after transfection for 24 h. The luciferase activity was assayed using the Dual-Luciferase Reporter Assay System (Promega, USA).

### The establishment and treatment of the POF model in vivo

SD female rats (five-week-old) purchased from The Experimental Animal Center of Jiangsu University were used for subsequent experiments after 7 days of acclimatization. Seventy-two rats were confirmed to have normal estrus cycles and randomly divided into four groups: WT, POF + PBS, POF + sEVs, and POF + si-YB-1 sEVs group. The establishment of POF rat model was accomplished by injection of CTX. The specific method was 50 mg/kg/d CTX injection for one day followed by 8 mg/kg/d CTX injection for 13 consecutive days (Yang et al. 2020a). Detailed animal information is provided in Supplementary Information: Animal protocol.

### Location of BMSCs-sEVs in vivo or vitro

In *vivo*, BMSCs-sEVs were stained with fluorochrome Dil (C1036, Beyotime, China) and injected into normal rats 6, 12, 24, 48, and 72 h later the lung, heart, liver, kidney, spleen, ovary, uterus of the rats and the location of BMSCs-sEVs in *vivo* detected via IVIS® Lumina LT Series III (PerkinElmer, USA) (wavelength = 550 nm). Repeat each experiment three times. In *vitro*, sEVs were incubated with 1 μM PKH26 (mini26, Sigma-Aldrich, USA) for 10 min at room temperature before unbound dye was removed by centrifugation. Subsequently, the labeled sEVs were added to the culture medium of KGN cells. Cells were fixed with 4% paraformaldehyde (P0099-100 ml, Beyotime, China) after 24 h of treatment. Following DAPI staining (C1006, Beyotime, China) Leica TCS SP5 II laser scanning confocal microscopy (Leica, GER) was used to detect the uptake of PKH26-labeled sEVs by KGN cells.

### Enzyme-linked immunosorbent assay

Serum was extracted by centrifugation from blood samples of rats collected from the heart. The ELISA kits (BPE30597, BPE30608, BPE30083, BPE30623, Lengton, China) are used to measure levels of some hormones in the serum, including FSH, E_2_, AMH, and LH.

### Immunohistochemistry (IHC)

The rat ovary tissues were fixed, embedded, and sectioned. the slides were incubated with the anti-YB-1 or anti-FOXO_3_ at 4 °C overnight. Next, slides were submerged in enhancers and secondary antibody for 1 h. Then DAB (P0203, Beyotime, China) was utilized for the chromogenic reaction. Counterstain slides with hematoxylin (ST2067-20 g, Beyotime, China). Then dehydrated, cleared, and evaluated. Finally, differential fields were randomly selected under the light microscope to capture images. The images were captured immediately under Leica DM4 B & DM6 B Upright Microscope and the image capture software was LAS Core.

### Assessment of reproductive potential

After two weeks of sEVs treatment, reproductive tests were performed. 6 rats in each group were mated with 3 sexually mature SD male rats. Mating success was determined by the presence or absence of sperm plugs. Each female rat was mated again after 3 weeks postpartum. Fertility levels, including pregnancy rate and number of offspring, were recorded for each group of rats. Female rats that had three consecutive positive sperm plugs but did not reproduce were ruled infertile.

### Statistical analysis

The data were based on three independent experiments and were displayed with mean ± standard deviation. GraphPad Prism software 8.0 for Windows (GraphPad Software, USA) was implicated in data analysis. Student’s t test was applied to evaluate the statistical significance between the two groups. Differences between two or more groups were analyzed using one-way ANOVA. P < 0.05 was defined as a significant difference (*P < 0.05; **P < 0.01; ***P < 0.001, ****P < 0.0001) in this study.

## Results

### YB-1 is the key protein of BMSCs-sEVs for the treatment of POF

**Fig. 1:**
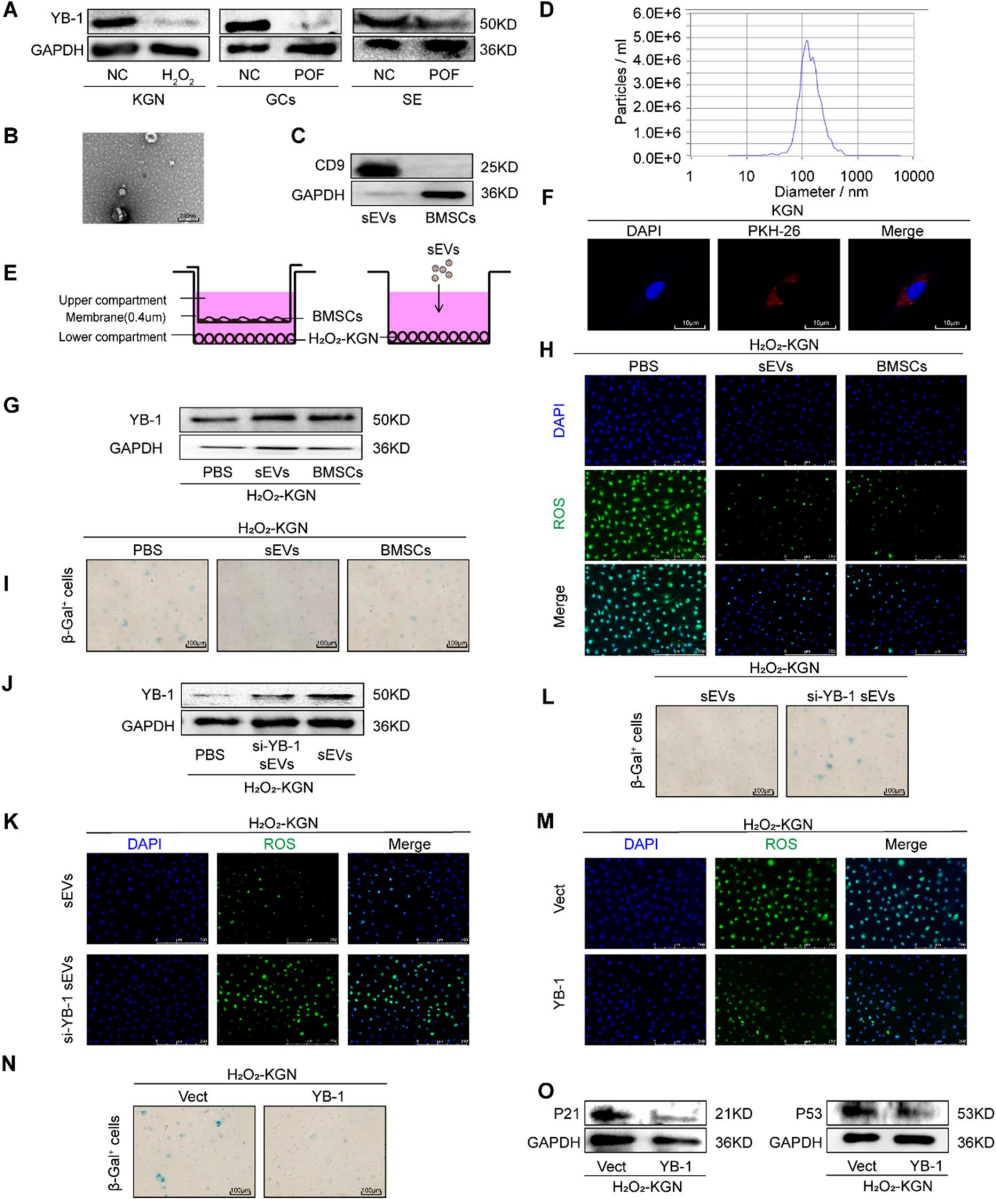
YB-1 is the key protein of BMSCs-sEVs for the treatment of POF. **A**. Western blot detected the expression of YB-1 in H_2_O_2_-KGN and the GCs or serum of patients with POF. **B**. Transmission electron microscopy was performed to observe the morphology of sEVs. **C**. Western blot was used to analyze the expression of exosome surface marker protein CD9. **D**. Size distribution of BMSCs-sEVs was assessed by a nanoparticle tracking analysis. **E**. Schematic diagram of H_2_O_2_-KGN cells cocultured with BMSCs and sEVs. **F**. KGN was incubated with PKH26-labeled sEVs, and the uptake of sEVs was observed under a fluorescent microscope. **G**. The difference expression of YB-1 in the H_2_O_2_ + PBS, H_2_O_2_ + sEVs and H_2_O_2_ + BMSCs groups was detected by Western blot. **H**. ROS in three experimental groups of KGN were assessed using DCFH-DA probes. The ROS‐positive apoptotic cells are indicated by green fluorescence. The nuclei (blue) were stained with DAPI. **I**. Immunohistochemistry staining of S-A-β-gal in the H_2_O_2_-KGN of the PBS, sEVs and BMSCs groups. **J**. The difference expression of YB-1 in the H_2_O_2_-KGN of the PBS, si-YB-1 sEVs and sEVs groups was detected by Western blot. **K**. The ROS level in the H_2_O_2_-KGN cocultured with sEVs and si-YB-1 sEVs were detected using DCFH-DA probes. **L**. Immunohistochemistry staining of S-A-β-gal in the H_2_O_2_-KGN cocultured with sEVs and si-YB-1 sEVs. **M**. ROS in oe-YB-1 group of H_2_O_2_-KGN were assessed using DCFH-DA probes. The ROS‐positive apoptotic cells are indicated by green fluorescence. The nuclei (blue) were stained with DAPI. **N**. Immunohistochemistry staining of S-A-β-gal in oe-YB-1 group of H_2_O_2_-KGN. **O**. Overexpressed YB-1 decreases protein levels of p21 and p53 in H_2_O_2_-KGN detected by western blot

To clarify the association between YB-1 and POF, we monitored the quantity of YB-1 in the GCs or serum of patients with POF and in a POF cell models, which was modeled as previously described (Liu et al. 2016). As shown in Fig. 1A and S1A-C, YB-1 was notably downregulated in the POF cell model and the GCs or serum of POF patients undergoing In Vitro Fertilization and Embryo Transfer (IVF-ET), implying a potential link between YB-1 and POF. Our previous study confirmed that BMSCs-sEVs could repair the decreased ovarian function caused by oxygen damage (Yang et al. 2020a); thus, we were curious to see if YB-1 was involved in this process. Therefore, we isolated and characterized sEVs from BMSCs. The sEVs showed a bilayer cup-shaped morphology (Fig. 1B), and expressed the sEVs marker protein CD9 (Fig. 1C). Moreover, a peak in particle size at 100 nm was detected by NTA (Fig. 1D). To determine the effects of BMSCs or sEVs, we employed a transwell non-contact culture system for BMSCs and H_2_O_2_-KGN cells and a contact coculture system for BMSCs-sEVs and POF cells (Fig. 1E). KGN exhibited efficient uptake of sEVs after 24 h (Fig. 1F). H_2_O_2_ was used to trigger premature senescence as previously reported (Burdon 1995; Chen et al. 2007; Toussaint et al. 2000). H_2_O_2_-treated KGN displayed a flattened and enlarged morphology and increased activity of S-A-β-gal, a widely used senescence marker (Fig. S1D-E). Western blot analysis revealed higher levels of other senescence markers such as p21 and p53, indicating the successful establishment of the POF model in *vitro* (Fig. S1F). Interestingly, BMSCs-sEVs led to an increase in YB-1 and reduced ROS generation, and improved the cellular senescence phenotype in H_2_O_2_-KGN cells (Fig. 1G-I) which was attenuated by YB-1 silencing in BMSCs (Fig. 1J-L and S1G). In addition, to demonstrate whether YB-1 is the active element responsible for senescence, YB-1 was overexpressed in the POF model in *vitro* (Fig. S1H). Direct overexpression of YB-1 effectively reduced senescence and oxidative stress levels (Fig. 1M-O and S1I-J). Taken together, it is clear from these data that YB-1 is the key player in BMSCs-sEVs-mediated alleviation of ROS-triggered cellular senescence in POF.

### YB-1 enhances MALAT1 RNA stability

**Fig. 2:**
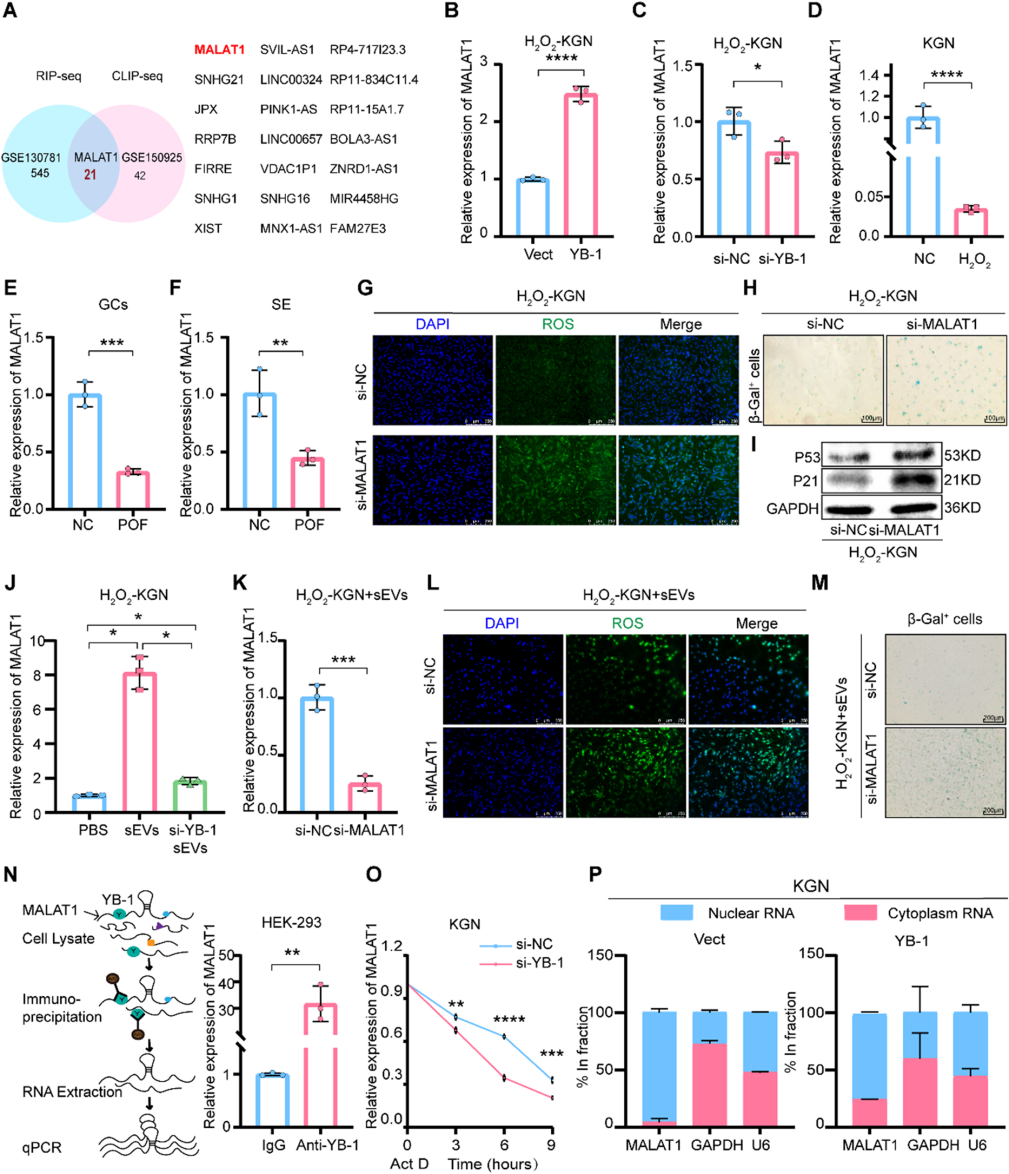
YB-1 enhances MALAT1 RNA stability. **A**. Venn diagram of LncRNA predicted bind to YB-1 in GSE130781 and GSE150925 database (http://www.ncbi.nlm.nih.gov/geo/), **B**-**C**. The expression of MALAT1 in the H_2_O_2_-KGN of oe-YB-1 (B) and si-YB-1 (C) groups was detected by qPCR. **D**. qPCR analysis of MALAT1 expression in H_2_O_2_-KGN. **E**–**F**. MALAT1 expression in the GCs (E) or serum (F) of healthy people and patients with POF was measured by qPCR. **G**. ROS in si-NC and si-MALAT1 of H_2_O_2_-KGN were assessed using DCFH-DA probes. The ROS‐positive apoptotic cells are indicated by green fluorescence. The nuclei (blue) were stained with DAPI. **H**. Immunohistochemistry staining of S-A-β-gal in si-NC and si-MALAT1 of H_2_O_2_-KGN groups. **I**. The difference expression of p21 and p53 in si-NC and si-MALAT1 of H_2_O_2_-KGN groups was detected by Western blot. **J**. The expression of MALAT1 in the H_2_O_2_-KGN of PBS, sEVs and si-YB-1 sEVs groups was detected by qPCR. K. qPCR analysis of MALAT1 expression in H_2_O_2_-KGN treated with si-MALAT1 and sEVs. **L**. ROS in si-NC and si-MALAT1 of H_2_O_2_-KGN + sEVs were assessed using DCFH-DA probes. The ROS‐positive apoptotic cells are indicated by green fluorescence. The nuclei (blue) were stained with DAPI. **M**. Immunohistochemistry staining of S-A-β-gal in si-NC and si-MALAT1 of H_2_O_2_-KGN + sEVs groups. **N**. YB-1 RIP was performed, and the enrichment of MALAT1 in YB-1 immunoprecipitation was assayed by qPCR analysis. IgG was used as negative control. **O**. The expression levels of MALAT1 in KGN cells treated with si-YB-1 were detected by qPCR at 0, 3, 6, and 9 h posttreatment with Actinomycin D. **P**. Cytoplasmic and nuclear fractions were extracted from KGN of Vector and oe-YB-1 groups and MALAT1 expression was analyzed by qPCR

To explore the possible mechanism by which YB-1 mediates ovarian repair, sequencing was performed for further analysis. Upon merging RIP-seq data (GSE130781) and CLIP-seq data (GSE150925), 21 candidate genes were identified as potential binding targets of YB-1 (Fig. 2A). Among them, when YB-1 was overexpressed, the expression profiles of 10 genes were changed, and MALAT1 was increased significantly (Fig. 2B-C). The correlation between MALAT1 and aging-related diseases should be considered (Ghafouri-Fard et al. 2021). The impact of MALAT1 on POF was characterized next. Remarkably, MALAT1 was downregulated in KGN cells treated with H_2_O_2_, and similar results were detected in serum and GCs derived from POF patients (Fig. 2D-F). Additionally, functional experiments confirmed that ROS levels were elevated (Fig. 2G), and S-A-β-gal activity and the level of p21 and p53 were increased in the si-MALAT1 group (Fig. 2H-I and 2SB). Notably, MALAT1 was significantly increased in H_2_O_2_-KGN cells after coculturing with BMSCs-sEVs, but this increase was reversed by YB-1 depletion in BMSCs-sEVs (Fig. 2J). Consistently, the MALAT1expression, ROS levels and S-A-β-gal activity were increased by MALAT1 depletion in H_2_O_2_-KGN cells after coculturing with BMSCs-sEVs (Fig. 2K-M).

To further define the interaction between MALAT1 and YB-1, we performed RIP in HEK-293 cells. The results demonstrated that MALAT1 was abundant in the RIP retrieved from the anti-YB-1 antibody group compared to that from the IgG group (Fig. 2N), supporting the notion that YB-1 directly interacted with MALAT1. The qPCR results revealed that the half-life of MALAT1 was shortened in a time-dependent manner when YB-1 was silenced (Fig. 2O). Interestingly, the subcellular fractionation analyses suggested that MALAT1 was mostly localized in the nuclei of KGN cells, while overexpression of YB-1 promoted the shuttling of MALAT1 to the cytoplasm (Fig. 2P). These results provide evidence that YB-1 enhances the RNA stability of MALAT1.

### MALAT1 functions as a ceRNA by directly sponging miR-211-5p

**Fig. 3:**
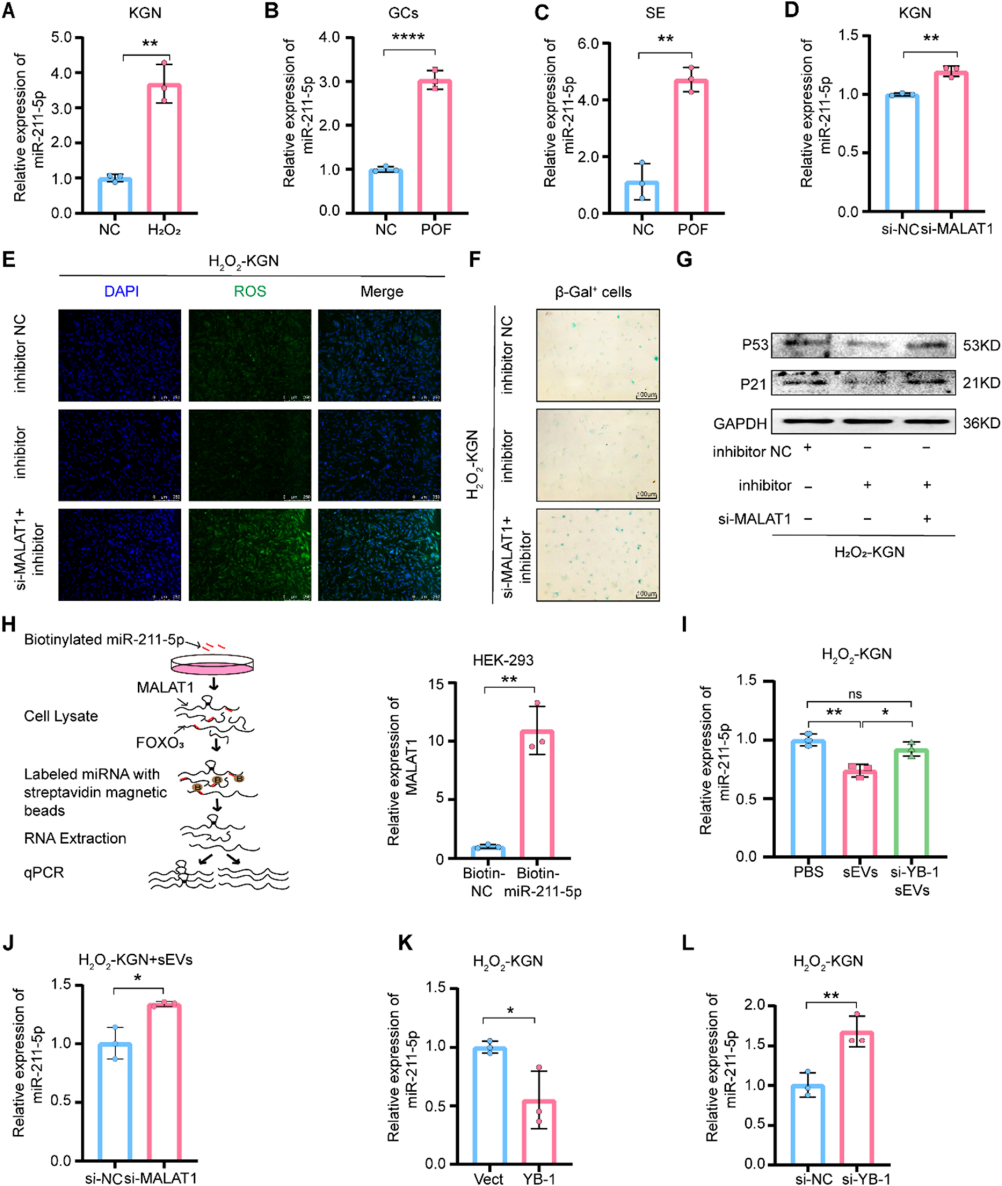
MALAT1 functions as a ceRNA by directly sponging miR‐211-5p. **A**. qPCR analysis of miR-211-5p expression in H_2_O_2_-KGN. **B**-**C**. miR-211-5p expression in the GCs (B) and serum (C) of healthy people and patients with POF was detected by qPCR. **D**. The expression levels of miR-211-5p in KGN cells treated with si-MALAT1 were measured by qPCR. **E**. ROS in three groups of H_2_O_2_-KGN were assessed using DCFH-DA probes. The ROS‐positive apoptotic cells are indicated by green fluorescence. The nuclei (blue) were stained with DAPI. **F**. Immunohistochemistry staining of S-A-β-gal in inhibitor NC, inhibitor and si-MALAT1 + inhibitor and groups of H_2_O_2_-KGN. **G**. The difference expression of p21 and p53 in the H_2_O_2_-KGN of three groups was detected by Western blot. **H**. Schematic of pull-down assay with biotinylated miRNA. Enrichment of MALAT1 in HEK-293 cells after pull-down assay with biotinylated miR-211-5p. **I**. The expression of miR-211-5p in the H_2_O_2_-KGN of PBS, si-YB-1 sEVs and sEVs groups was detected by qPCR. **J**. qPCR analysis of miR-211-5p expression in H_2_O_2_-KGN + sEVs cells treated with si-MALAT1. **K**-**L**. The expression levels of miR-211-5p in H_2_O_2_-KGN cells treated with oe-YB-1 (K) and si-YB-1 (L) were measured by qPCR

Since one of the classical mechanisms of lncRNA is the ceRNA network, we further sought to investigate whether MALAT1 could promote ceRNA network progression. Previous studies have shown that there is a strong link between miR-211-5p and aging-related diseases (Fan et al. 2016). In addition, the starBase (https://rnasysu.com/encori/) provides the information that miR-211-5p is a potential downstream target of MALAT1. Then, we first examined the miR-211-5p expression and found increases in miR-211-5p in the POF cell model and patients (Fig. 3A-C). Additionally, silencing MALAT1 increased miR-211-5p expression (Fig. 3D). Interestingly, knocking down MALAT1 inhibition significantly elevated ROS levels and S-A-β-gal activity in H_2_O_2_-KGN cells while anti-miR-211-5p reduced ROS accumulation and S-A-β-gal activity (Fig. 3E-F), as well as p53 and p21 expression (Fig. 3G and Fig. S3A-B), supporting the hypothesis that MALAT1 inhibits cell senescence by blocking miR-211-5p activity. Furthermore, the direct interaction between MALAT1 and miR-211-5p was demonstrated (Fig. 3H). Consistently, in the cocultivation system, silencing YB-1 in BMSCs-sEVs in a cocultivation system or silencing MALAT1 in H_2_O_2_-KGN directly elevated miR-211-5p content in H_2_O_2_-KGN cells (Fig. 3I-J). Likewise, YB-1 overexpression decreased the quantity of miR-211-5p, while YB-1 silencing increased miR-211-5p level in H_2_O_2_-KGN cells (Fig. 3K-L). These results suggest that MALAT1 could perform ceRNA functions by directly sponging miR‐211-5p.

### FOXO_3_ is a direct downstream target of miR-211-5p

**Fig. 4:**
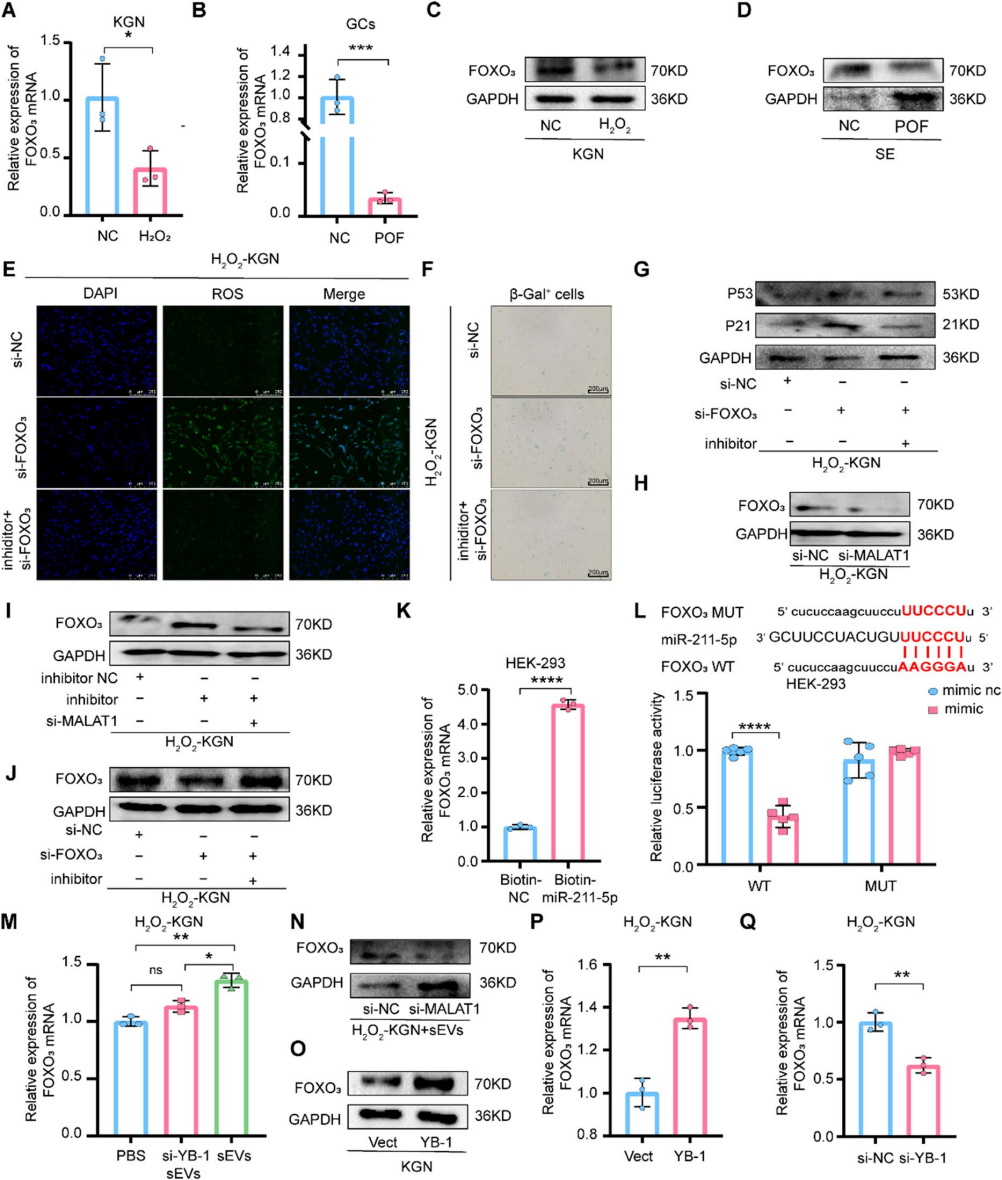
FOXO_3_ is a direct downstream target of miR-211-5p. **A**. qPCR analysis of FOXO_3_ mRNA expression in H_2_O_2_-KGN cells. **B**. The relative FOXO_3_ mRNA expression was measured using qPCR in the GCs of healthy people and patients with POF. **C**-**D**. FOXO_3_ protein expression level was detected in H_2_O_2_-KGN cells (C) or the serum (D) of healthy people and patients with POF by western blot. **E**. ROS in si-NC, si-FOXO_3_ and si-FOXO_3_ + inhibitor of H_2_O_2_-KGN were assessed using DCFH-DA probes. The ROS‐positive apoptotic cells are indicated by green fluorescence. The nuclei (blue) were stained with DAPI. **F**. Immunohistochemistry staining of S-A-β-gal in si-NC, si-FOXO_3_ and si-FOXO_3_ + inhibitor groups. **G**. The difference expression of p21 and p53 in the KGN of three groups was detected by western blot. **H**-**J**. The relative FOXO_3_ protein expression was measured using western blot in different groups of H_2_O_2_-KGN. **K**. Enrichment of FOXO_3_ mRNA in HEK-293 cells after pull-down assay with biotinylated miR-211-5p. **L**. Wild type and mutant FOXO_3_ sequences were cloned into the pGL4 luciferase reporter vector and co-transfected with miR-211-5p into HEK-293 cells followed by dual luciferase assay. **M**. The expression of FOXO_3_ mRNA in the H_2_O_2_-KGN of PBS, si-YB-1 sEVs and sEVs groups was detected by qPCR. **N**. Western blot analysis of FOXO_3_ expression in H_2_O_2_-KGN + sEVs cells treated with si-MALAT1. **O**. Western blot detection of FOXO_3_ protein expression in KGN delivering oe-YB-1. **P**-**Q**. The expression levels of FOXO_3_ mRNA in H_2_O_2_-KGN cells treated with oe-YB-1 (P) and si-YB-1 (Q) were measured by qPCR

It is well known that FOXO have been established as longevity genes (Morris et al. 2015; Singh et al. 2019) (specifically, FOXO_3_) and promoters of senescence (Baar et al. 2017). In addition, the downstream targets of MALAT1, including the miR-211-5p-FOXO_3_ network, were predicted using the starBase (https://rnasysu.com/encori/). Then, they were selected to establish our ceRNA network. Decreases in FOXO_3_ expression were observed in the POF cell model and patients (Fig. 4A-D and Fig. S4A-B). Additionally, silencing FOXO_3_ significantly elevated ROS levels and S-A-β-gal activity in H_2_O_2_-KGN cells, as well as p53 and p21 expression. However, these effects were attenuated by anti-miR-211-5p (Fig. 4E-G). Likewise, silencing MALAT1 decreased FOXO_3_ (Fig. 4H and Fig. S4C), and anti-miR-211-5p transfection in MALAT1- silenced cells partially increased FOXO_3_ expression (Fig. 4I and Fig. S4D). Similarly, the simultaneous silencing of FOXO_3_ and miR-211-5p caused upregulation of FOXO_3_ (Fig. 4J). To further validate the MALAT1/miR-211-5p/FOXO_3_ pathway, the interaction between FOXO_3_ and miR-211-5p was detected by a pull-down assay with biotinylated miRNA (Fig. 4K). Furthermore, once the FOXO_3_ binding motif of “AAGGGA” of miR-211-5p was mutated as mutant FOXO_3_, luciferase activity was not suppressed, compared with decreased activity in the presence of the wild-type miR-211-5p binding motif (Fig. 4L). Consistently, in the cocultivation system, silencing YB-1 in BMSCs-sEVs or silencing MALAT1 in H_2_O_2_-KGN directly increased FOXO_3_ (Fig. 4M-N). Likewise, YB-1 overexpression increased the level of FOXO_3_, while YB-1 silencing decreased FOXO_3_ expression (Fig. 4O-Q and Fig. S4E). These results collectively suggest that YB-1 stabilized MALAT1 regulates GCs senescence via the miR-211-5p/FOXO_3_ network.

### The therapeutic effect of YB-1 sEVs on POF

**Fig. 5:**
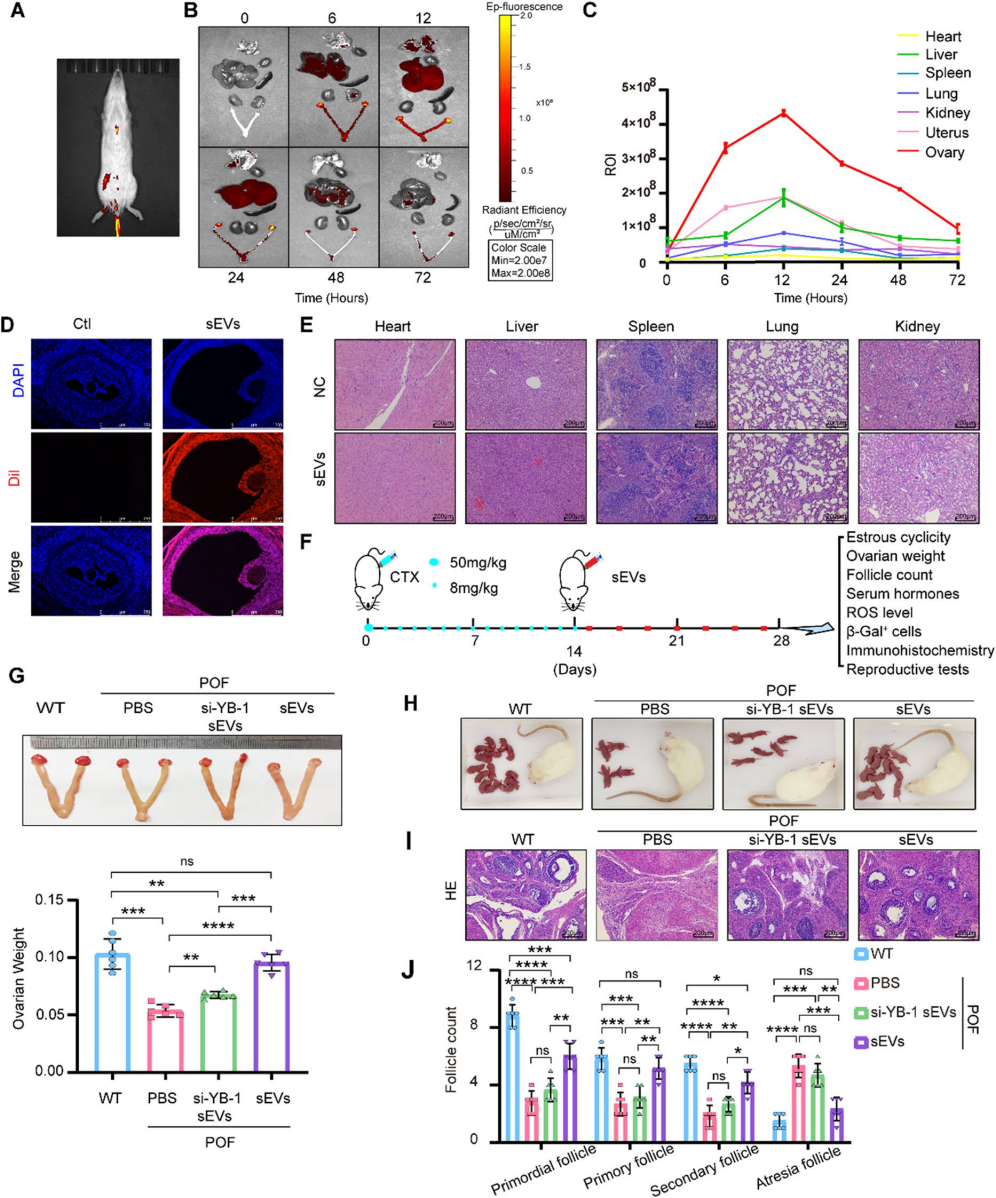
Therapeutic effect of YB-1 sEVs on POF. **A**. Representative IVIS images of rats injected with 100 μl PBS, 100 μg (in 100 μl) Dil-labeled sEVs via the tail vein. IVIS imaging was performed 24 h after injection. **B**-**C**. Ex *vivo* fluorescence imaging analysis of the distribution of the Dil-labeled sEVs in different organs, including the ovary, liver, spleen, heart, kidney, uterus and lung (B). Quantification of the fluorescence signal intensity in different organs (C). IVIS imaging was performed 6, 12, 24, 48, and 72 h after injection. **D**. Histopathological examination of different organs of rat injected with sEVs. **E**. Representative fluorescence microscopic images of the localization of Dil-labeled sEVs in ovary. Rat was injected with 100 μl Dil-labeled sEVs via tail vein and sacrificed 24 h after injection. **F**. Flow chart of the animal experiment. **G**. The weights of ovaries in the WT, POF + PBS and POF + si-YB-1 sEVs and POF + sEVs groups were measured. **H**. Representative outcomes of different groups. **I**. Histopathological examination of the ovaries in different groups. **J**. Primordial follicles, primary follicles, secondary follicles, and atretic follicles were observed in each group of rats

To elucidate the repair effect of YB-1 sEVs on ovarian function in POF in *vivo*, we first determined the targeting capacity of sEVs. First, the POF model was established with CTX, with 50 mg/kg for one day followed by 8 mg/kg/d CTX injection for 13 consecutive days (Liu et al. 2016). Ovary weight, counts of follicles at various phases of growth, and normal estrous cyclicity were notably diminished in the POF group. The biodistribution of sEVs was investigated in rats utilizing noninvasive near-infrared fluorescence. Fluorescence signals were detected around the liver, lung, and ovary after intravenous injection (Fig. 5A-B). Moreover, the sEVs concentration in major organs was also investigated. As shown in Fig. 5C, sEVs had a maximum concentration in the ovary, followed by the liver and uterus at 12 h, after which the signal began to decline. The Dil-signal was detected in the follicles, which showed efficient uptake of sEVs (Fig. 5D), while sEVs could not lead to substantial damage to the heart, liver, spleen, lung, or kidney (Fig. 5E). As shown in Fig. 5F, the POF model was induced by CTX and was treated with sEVsonce every 2 days for 2 weeks. Under BMSCs-sEVs treatment, the ovarian morphology and follicle count were obviously restored, and ovarian weight, pregnancy rate, number of offspring and regular estrous cyclicity were significantly increased, serum hormone levels showed significant improvement in the BMSCs-sEVs transplant group after chemotherapy. However, the therapeutic effect (restoration of ovarian morphology, follicle count, ovarian weight, fertility (pregnancy rate, number of offspring), estrous cyclicity, and serum hormones) of sEVs was significantly attenuated by YB-1 depletion (Fig. 5G-J and Fig. S5A-E).

### SEVs improve the symptoms of POF via the sEVs/YB-1/MALAT1/miR-211-5p/FOXO_3_ axis

**Fig. 6:**
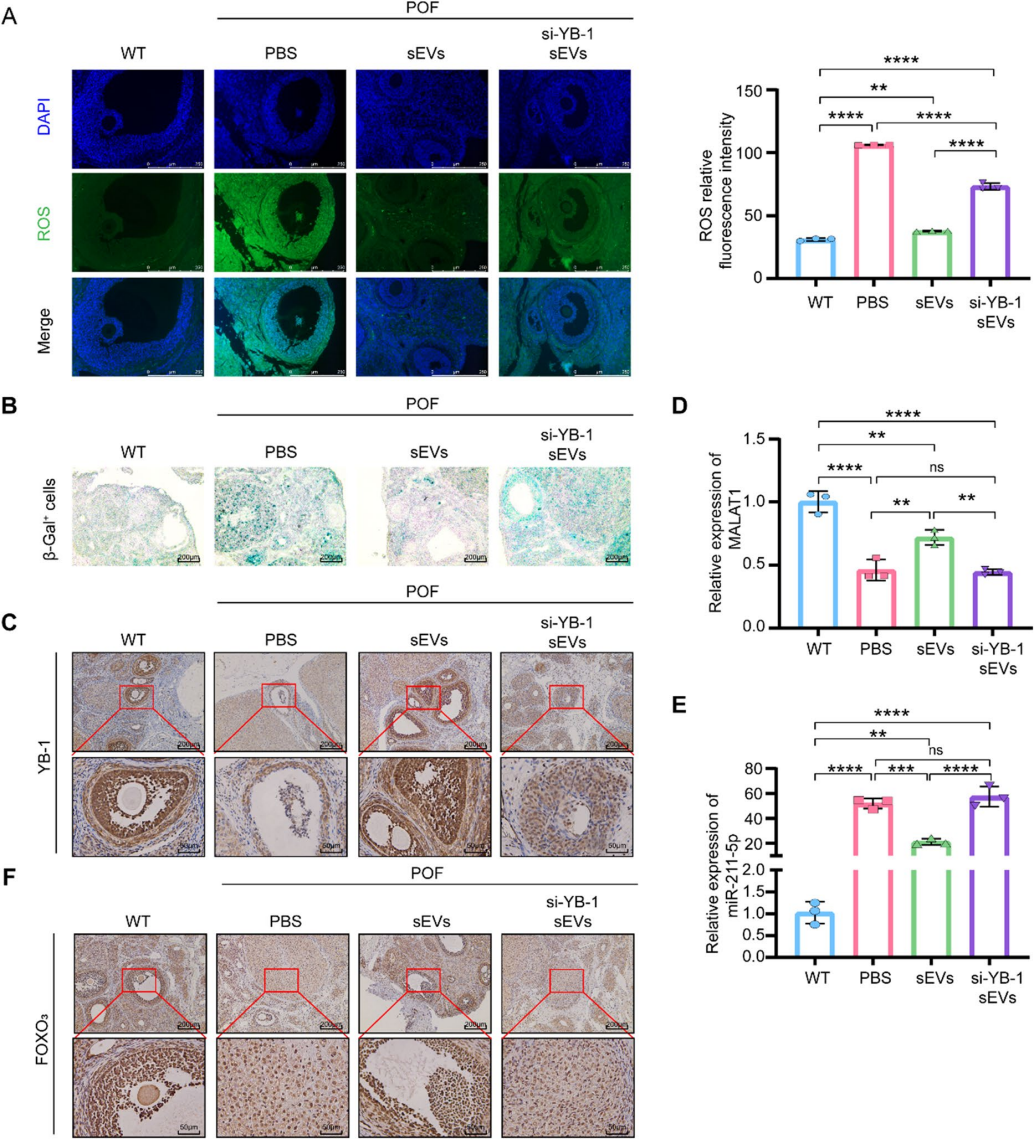
SEVs improve the symptoms of POF via sEVs/YB-1/MALAT1/miR-211-5p/FOXO_3_ axis. **A**. ROS in different treatment groups were assessed using DCFH-DA probes. The ROS‐positive apoptotic cells are indicated by green fluorescence. The nuclei (blue) were stained with DAPI. **B**. Immunohistochemistry staining of S-A-β-gal in different treatment groups. **C**. Enlarged images from representative immunohistochemistry photographs of YB-1 in different treatment groups. **D**. qPCR analysis of MALAT1 expression in different treatment groups. **E**. qPCR analysis of miR-211-5p expression in different treatment groups. **F**. Enlarged images from representative immunohistochemistry photographs of FOXO_3_ in different treatment groups

**Fig. 7:**
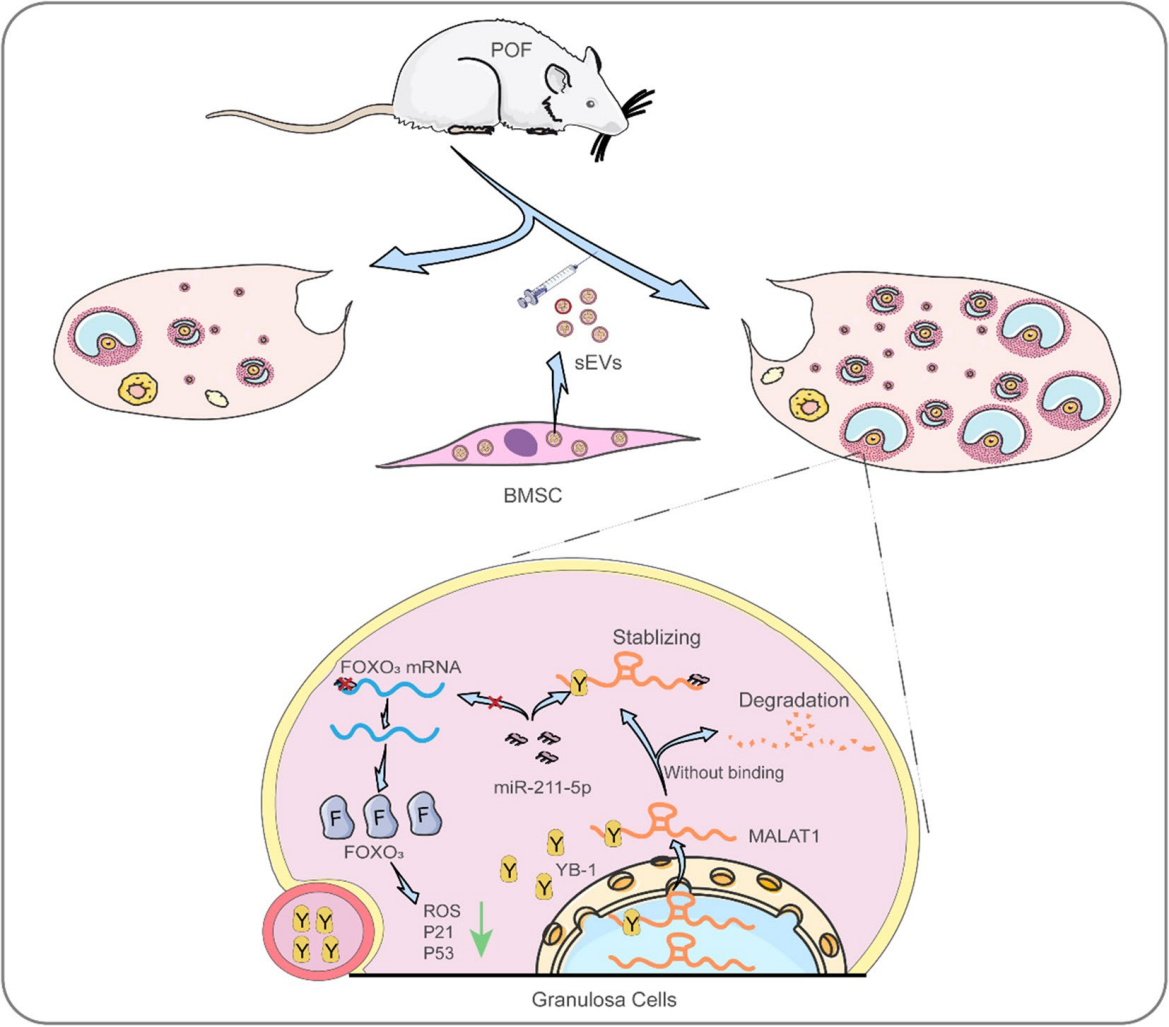
Schematic summary of the critical role of YB-1 in GCs expression and function

Next, to verify the intimate connection of each factors in the sEVs/YB-1/MALAT1/miR-211-5p/FOXO_3_ axis in *vivo*, we characterized their correlations in POF rats. First, we confirmed that the ROS level and S-A-β-gal were increased in the si-YB-1 sEVs group (Fig. 6A-B). Likewise, YB-1, MALAT1, and FOXO_3_ were decreased in the si-YB-1 sEVs group in *vivo*, accompanied by an increase in miR-211-5p (Fig. 6C-F). Thus, our experimental results indicate that sEVs could also significantly improve the symptoms of POF through the sEVs/YB-1/MALAT1/miR-211-5p/FOXO_3_ pathway (Fig. 7).

## Discussion

The prevalence of POF in women is 1% (Pal and Santoro 2002), and CTX treatment is among the causes of POF (Chand et al. 2010). Therefore, management of degenerative ovarian changes and improvement ovarian function are crucial to maintaining female fertility (Park et al. 2021). Reducing ROS generation, improving antioxidant capacities, and repairing oxidative stress-induced GCs senescence are effective treatment measures (Schieber and Chandel 2014), for that, MSCs transplantation is thought to be a front-end therapy, and the treatment functions of MSCs could, at least in part, be mediated by EXOs (Toh et al. 2018), which are biocompatible, nonimmunogenic and have low tumorigenicity and embolization compared to MSCs transplantation, thus allowing allotransplantation, and has been considered as a novel method to alleviating age-related fertility retardation in women (Pegtel and Gould 2019; Toh et al. 2018). Furthermore, as a shuttle vehicle between cells, the therapeutic effect of EXOs could be enhanced by changing its contents (Liu et al. 2021; Qu et al. 2022), that eventually lead to the importance of studying the global gene expression involving in the pathogenesis POF.

In the course of our experiments, we found that YB-1 expression was markedly decreased in the POF rats and cell model and increased by MSCs-sEVs transplantation, importantly, decreased YB-1 was detected in the serum and GCs of POF patients as well. Thus, we examined whether YB-1 had a critical effect in mediating MSCs-sEVs based treatment for POF. As expected, the therapeutic effect of MSCs-sEVs on oxidative damage-induced ovarian dysfunction and GCs senescence was significantly counteracted in *vitro* and in *vivo* by YB-1 depletion. Next, the secret behind the anti-oxidative stress effect of YB-1 was explored. As a broad-specificity RBP, YB-1 has been shown to participate in many aspects of RNA metabolism by recognizing and binding to RNA (Lyabin et al. 2014). By querying the GEO database, we found the RIP-seq data (GSE130782) and CLIP-seq data (GSE150925) of YB-1 and selected MALAT1, a lncRNA, which was overexpressed in multiple cancers and deeply involved in several physiological processes (Paronetto et al. 2020), including cellular senescence and healthy aging from the intersection of both and verified their direct binding effect. Mechanistic investigations further demonstrated that YB-1 was capable of increasing the steady-state levels of MALAT1 and facilitated the shuttling of MALAT1 from the nucleus to the cytoplasm. Previous studies demonstrated that Yps, a Drosophila Y-box protein, modulates the subcellular localization of Osk mRNA in the Drosophila ovary by antagonizing the osk mRNA localization enhancer Orb(Christerson and McKearin 1994; Mansfield et al. 2002; Wilhelm et al. 2000). In addition, YB-1 binds to the influenza virus single-stranded RNA genome (in the form of the vRNP complex) and exports it from the nucleus (Kawaguchi et al. 2012).

Previous research suggested that YB-1 specifically binds to a CA(U/C)C consensus sequence, and the second adenosine (A2) and the fourth cytidine (C4) are key to YB-1 recognition (Budkina et al. 2020). We further explored the YB-1 and MALAT1 binding sites by comparing the sequences, and found that MALAT1 contained two sequences that predominantly bind to YB-1. These sequences are, respectively, “CCUGCGGC” and “ACCAGCCU”, located at 65506163–65506170 and 65502668–65502675 on chromosome 11. In addition, by analyzing the RIP-seq data (GSE130782) and CLIP seq data (GSE150925), we found YB-1 combined with the “ACUAAAACCAACUUAAACCAG” sequence of MALAT1, which is located at 65505792–65505812 on chromosome 11(Fig. S2). Although specific binding sites between YB-1 and MALAT1 requires further verification, the existence of this potential binding sequence makes it more credible that there is a biological binding between them two.

Further, based on sequencing data, bioinformatics analysis and loss/gain-of function method, we identified miR-211-5p/FOXO_3_ was the direct target of MALAT1 in regulation of oxidative damage in GCs. Consistently, female mice that were generated with disruptions of FOXO_3_ exhibited age-dependent infertility and had abnormal ovarian follicular growth (Hosaka et al. 2004). Taking together, we exposed a novel effect of YB-1 in MSCs-sEVs-mediated ovarian function repair via adaptive regulation of the MALAT1/miR-211-5p/FOXO_3_ signaling pathway. As research expands, more and more studies are suggesting that the therapeutic role of sEVs in POF is highly anticipated. However, there are still many challenges to overcome for the use of EVs in clinical trials for POF. For example, the large-scale production of sEVs is limited by the complexity of the preparation process and the immaturity of the isolation and the efficiency of sEVs in targeting ovaries needs to be further improved. In addition, although sEVs have been tested in animal models of POF, their therapeutic efficacy and safety in humans remain unclear. Once the above challenges are overcome, sEVs will show great potential in the treatment of POF.

## Conclusion

In conclusion, our study demonstrated that BMSCs-sEVs treatments were useful in *vitro* and in *vivo* and had a critical therapeutic role in the recovery of ovarian functions in a POF rat model by delivering YB-1. We provided evidence that MALAT1 was stabilized by interaction with YB-1 and served as a miR-211-5p sponge to reverse cell senescence, increase FOXO_3_ expression and repair ovarian function. Hence, this research not only sheds new light on the therapeutic mechanisms based on sEVs/YB-1/MALAT1/miR-211-5p/FOXO_3_ but also provides a new option for treating POF.

## Supplementary Information

Below is the link to the electronic supplementary material.

Supplementary file1 (DOCX 1380 KB)

## Data Availability

No datasets were generated or analysed during the current study.

## Abbreviations

POF: Premature ovarian failure
BMSCs-sEVs: Bone mesenchymal stem cells derived small extracellular vesicles
YB-1: Y-box binding protein
CTX: Cyclophosphamide
ROS: Reactive Oxygen Species
S-A-β-gal: Senescence-associated β-galactosidase
KGN: A human granulosa-like tumor cell line
qPCR: Real-time Quantitative PCR
MALAT1: Metastasis associated lung adenocarcinoma transcript 1
RIP: RNA immunoprecipitation
GCs: Granulosa cells
ceRNA: Competing endogenous RNA
EV: Extracellular vesicle
IVF-ET: In Vitro Fertilization and Embryo Transfer
CLIP: Cross-linking immunoprecipitation
HEK-293 cells: Human Embryonic Kidney 293 cells
IVIS: Spectrum in *vivo* imaging system
RBP: RNA-binding protein
3′UTRs: 3′Untranslated regions
IHC: Immunohistochemistry
HE: Hematoxylin and eosin
