# Supplementary material for: Exosomal YB-1 facilitates ovarian restoration by MALAT1/miR-211-5p/FOXO3 axis
Source: Cell Biol Toxicol. 2024 May 3;40(1):29. doi: 10.1007/s10565-024-09871-8 (PMC11068691; doi:10.1007/s10565-024-09871-8)
Supplement: Supplementary file 1 — Supplementary file1 (DOCX 1380 KB) [file 10565_2024_9871_MOESM1_ESM.docx]

**Supplementary Information**

**Table. S1: The primer sequences of MALAT1 miR-211-5p and FOXO_3_.**

| Hsa-MALAT1 F | TGGAATTTGGAGGGATGGGAGGAG |
| --- | --- |
| Hsa-MALAT1 R | ACTTGCCAACAGAACAGACAGACC |
| Hsa-FOXO_3_ F | AGCCGAGGAAATGTTCGTC |
| Hsa-FOXO_3_ R | CCTTATCCTTGAAGTAGGGCAC |
| Hsa-miR-211-5p | CTTCCCTTTGTCATCCTTCGCCT |
| Rno-lncMALAT1 F | GGGAGTGGTCTTAACAGGGAGGAG |
| Rno-lncMALAT1 R | AACAGCATAGCAGTACACGCCTTC |
| Rno-miR-211-5p | TTCCCTTTGTCATCCTTTGCCT |

**Animal protocol**

| **Group**  **Day** | WT (n=21) | POF+PBS (n=17) | POF+sEVs (n=17) | POF+si-YB-1 sEVs (n=17) |
| --- | --- | --- | --- | --- |
| 1 | 0.9% normal saline  (n=21) | CTX 50mg/kg score(n=17) | CTX 50mg/kg score(n=17) | CTX 50mg/kg score(n=17) |
| 2-6 | 0.9% normal saline  (n=21) | CTX 8mg/kg score(n=17) | CTX 8mg/kg score(n=17) | CTX 8mg/kg score(n=17) |
| 7 | 0.9% normal saline(n=18)  Serum hormones  (n=3) | CTX 8mg/kg score(n=16)  Serum hormones  (n=1) | CTX 8mg/kg score(n=16)  Serum hormones  (n=1) | CTX 8mg/kg score(n=16)  Serum hormones  (n=1) |
| 8-13 | 0.9% normal saline(n=18) | CTX 8mg/kg score(n=16) | CTX 8mg/kg score(n=16) | CTX 8mg/kg score(n=16) |
| 14 | 0.9% normal saline(n=15)  Estrous cyclicity  ovarian weight  Follicle count  Serum hormones  ROS level  Immunohistochemistry  (n=3) | CTX 8mg/kg score(n=15)  Estrous cyclicity  ovarian weight  Follicle count  Serum hormones  ROS level  Immunohistochemistry  (n=1) | CTX 8mg/kg score(n=15)  Estrous cyclicity  ovarian weight  Follicle count  Serum hormones  ROS level  Immunohistochemistry  (n=1) | CTX 8mg/kg score(n=15)  Estrous cyclicity  ovarian weight  Follicle count  Serum hormones  ROS level  Immunohistochemistry  (n=1) |
| 15,17,19 | PBS (n=15) | PBS (n=15) | sEVs (n=15) | Si-YB-1 sEVs (n=15) |
| 21 | PBS (n=12)  Serum hormones  (n=3) | PBS (n=12)  Serum hormones  (n=3) | sEVs (n=12)  Serum hormones  (n=3) | Si-YB-1 sEVs (n=12)  Serum hormones  (n=3) |
| 23，25，27 | PBS(n=12) | PBS(n=12) | sEVs (n=12) | Si-YB-1 sEVs (n=12) |
| 28 | Estrous cyclicity  ovarian weight  Follicle count  Serum hormones  ROS level  β-Gal+ cells  Immunohistochemistry  (n=6)  Reproductive tests  (n=6) | Estrous cyclicity  ovarian weight  Follicle count  Serum hormones  ROS level  β-Gal+ cells  Immunohistochemistry  (n=6)  Reproductive tests  (n=6) | Estrous cyclicity  ovarian weight  Follicle count  Serum hormones  ROS level  β-Gal+ cells  Immunohistochemistry  (n=6)  Reproductive tests  (n=6) | Estrous cyclicity  ovarian weight  Follicle count  Serum hormones  ROS level  β-Gal+ cells  Immunohistochemistry  (n=6)  Reproductive tests  (n=6) |

**Fig. S1**


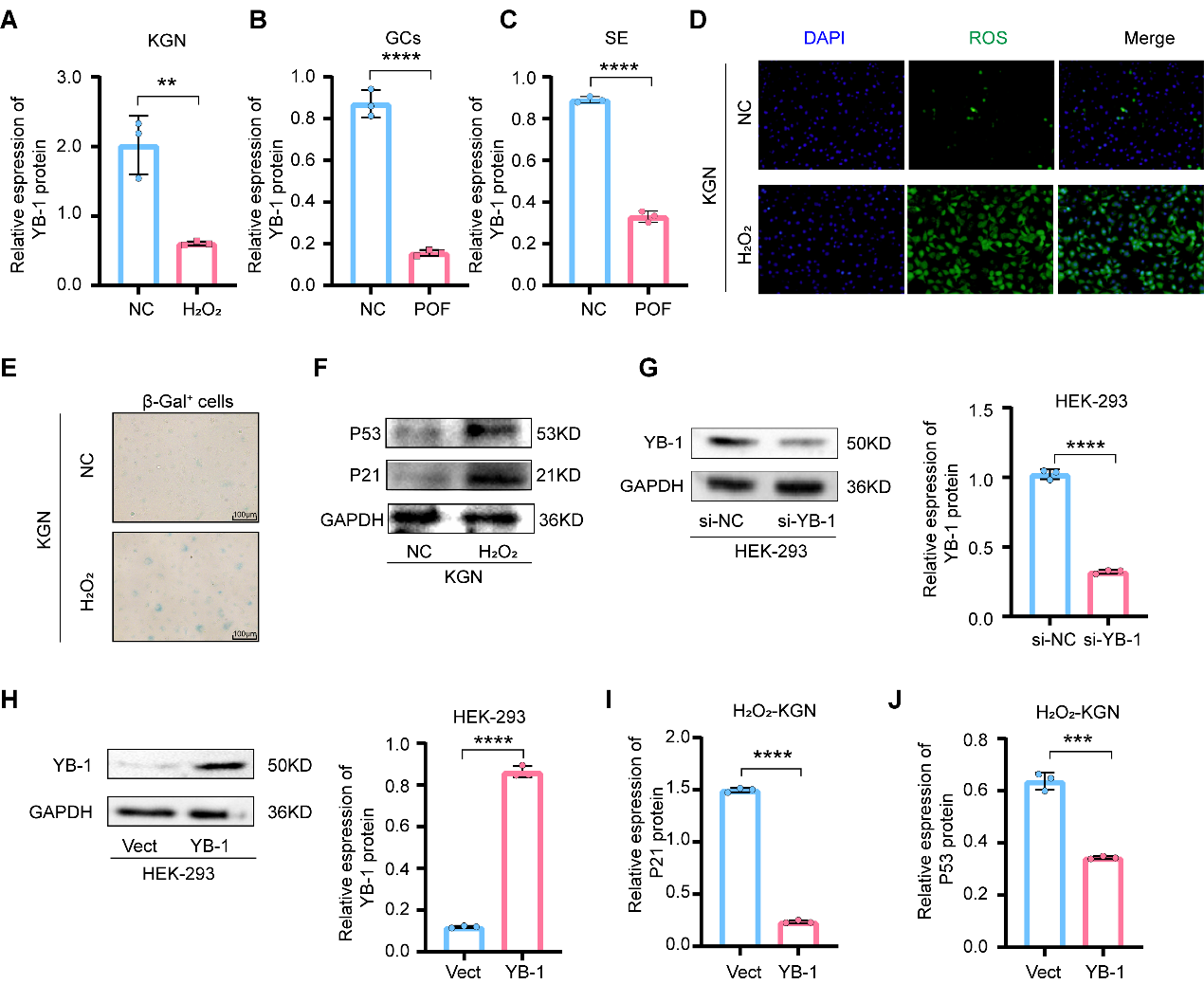


A-C. Quantification analysis of the protein level of YB-1 in relation to GAPDH in H_2_O_2_-KGN (A) and the GCs (B) and serum (C) of POF patients. D. The ROS level were detected using DCFH-DA probes in the H_2_O_2_-KGN. E. Immunohistochemistry staining of S-A-β-gal in the KGN treated with H_2_O_2_. F. Protein levels of p21 and p53 in H_2_O_2_-KGN detected by western blot. G-H. YB-1 protein was detected by western blot of lysates from H_2_O_2_-KGN treated with negative control and si-YB-1 (G) or oe-YB-1 (H). Quantification of western blot bands was conducted by ImageJ. I-J. protein levels of p21 (I) and p53 (J) in H_2_O_2_-KGN detected by western blot. Quantification of western blot bands was conducted by ImageJ.

**Fig. S2**


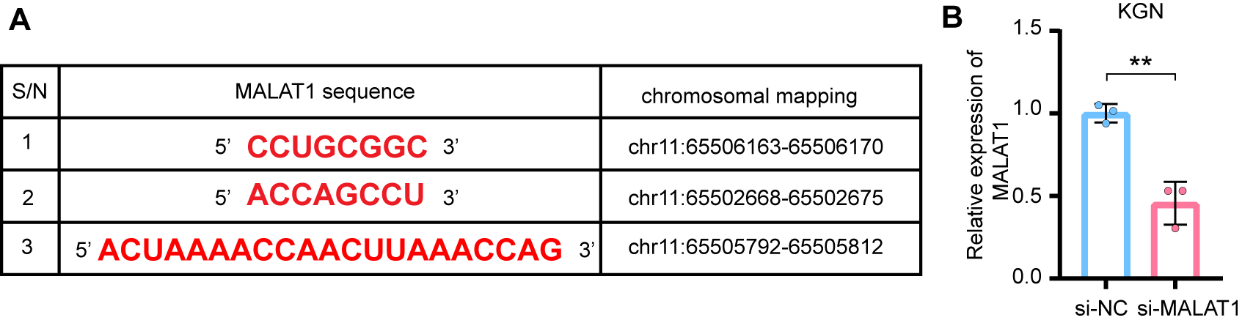


A. The potential binding sites between YB-1 and MALAT1. 1-2: the MALAT1 contains two sequences which were predominantly bind sites to YB-1 as previous research shows. 3: The sequence of MALAT1 which was forecasted by biological information combined with YB-1. B. The expression levels of MALAT1 in KGN cells treated with si-MALAT1.

**Fig. S3**


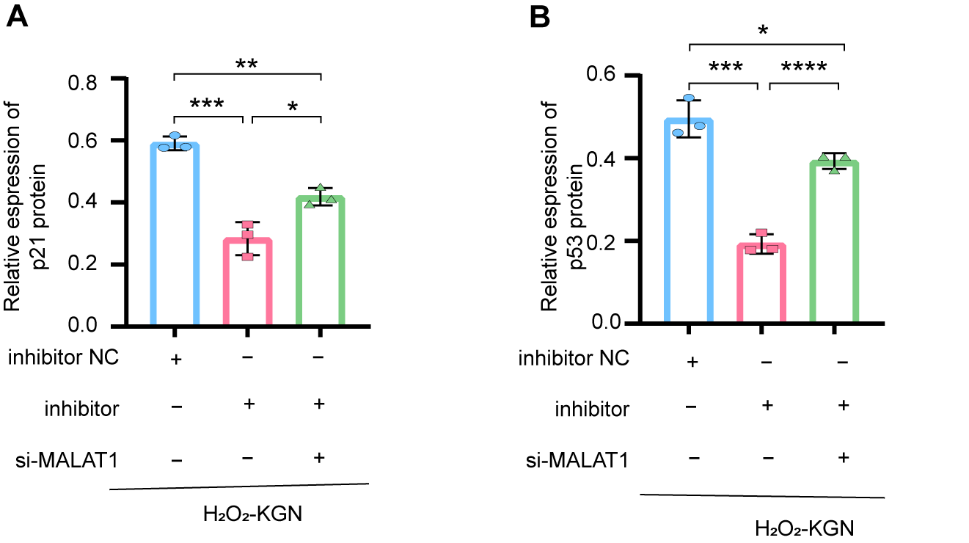


A-B. The differential expression of p21 (A) and p53 (B) in the KGN of the inhibitor NC, inhibitor, and si-MALAT1+ inhibitor was detected by western blot.

**Fig. S4**


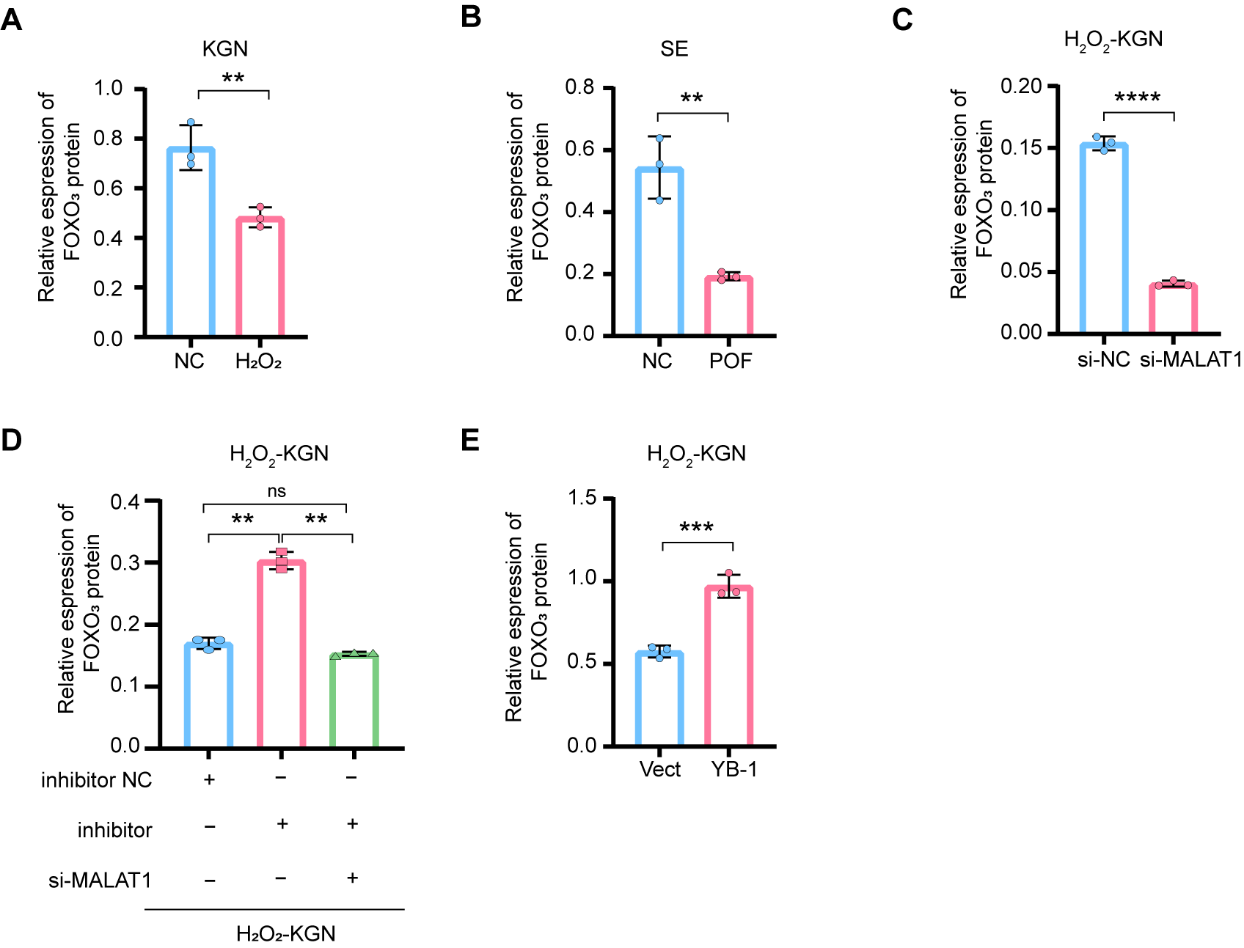


A. Quantification analysis of the protein level of FOXO_3_ in relation to GAPDH in KGN treated with H_2_O_2_. B. Quantification analysis of the protein level of FOXO_3_ in the SE of healthy people and patients with POF. C. The expression levels of FOXO_3_ protein in H_2_O_2_-KGN cells treated with si-MALAT1. D. Quantification analysis of the protein level of FOXO_3_ in relation to GAPDH in H_2_O_2_-KGN of inhibitor NC, inhibitor, and si-MALAT1+inhibitor groups. E. Quantification analysis of the protein level of FOXO_3_ in relation to GAPDH in H_2_O_2_-KGN treated with oe-YB-1.

**Fig. S5**

_
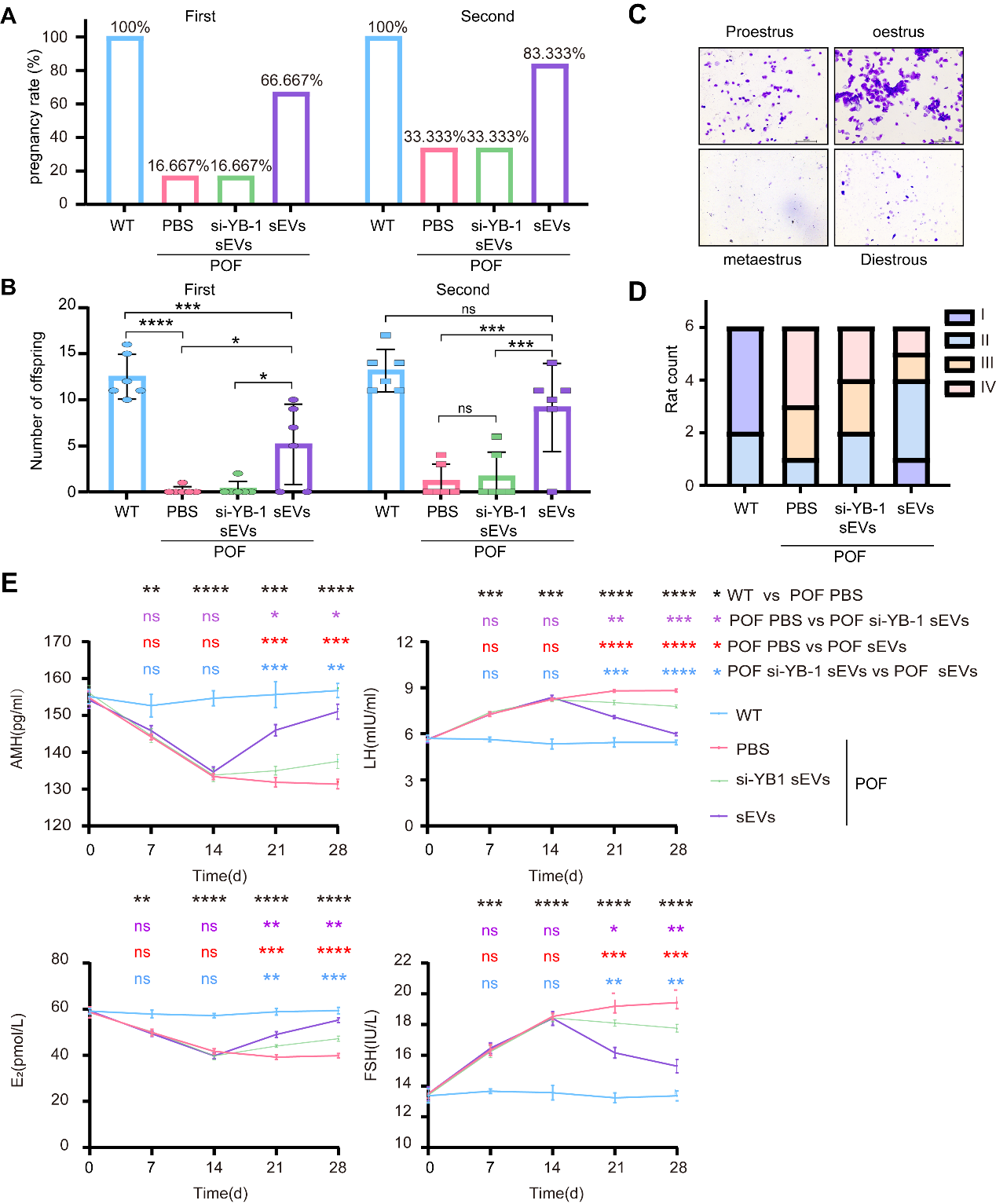
_

A. Pregnancy rate of normal rats and four treatment groups. B. Number of offspring in normal rats and four treatment groups. C-D. Normal estrous cycles: 1, proestrus; 2, estrus; 3, metestrus; and 4, diestrus (C). The total number of rats from each group was categorized into various estrous patterns (I–IV). I, normal; II, regular cycles with shortened estrus; III, irregular cycles with prolonged diestrus and normal or prolonged estrus; and IV, no cyclicity. The total number of rats from each group were categorized into various estrous patterns (I–IV) (D). E. Trends for the average levels of FSH, LH, AMH, and E_2_. Comparison of the AMH, E_2_, FSH, and LH levels between groups at different times. The “ns” (P > 0.05) indicates no statistically significant difference. *P < 0.05, **P < 0.01, and ***P < 0.001 for all figures. Statistical significance was determined using two-tailed t-tests for two groups and ANOVA for multiple comparisons. All values are means ± SD.
